# Prosaposin Deficiency and Saposin B Deficiency (Activator-Deficient Metachromatic Leukodystrophy): Report on Two Patients Detected by Analysis of Urinary Sphingolipids and Carrying Novel PSAP Gene Mutations

**DOI:** 10.1002/ajmg.a.32712

**Published:** 2009-03-06

**Authors:** Ladislav Kuchař, Jana Ledvinová, Martin Hřebíček, Helena Myšková, Lenka Dvořáková, Linda Berná, Petr Chrastina, Befekadu Asfaw, Milan Elleder, Margret Petermöller, Heidi Mayrhofer, Martin Staudt, Ingeborg Krägeloh-Mann, Barbara C Paton, Klaus Harzer

**Affiliations:** 1 Charles University in Prague, 1st Medical Faculty, Institute of Inherited Metabolic Disorders of 1st Faculty of Medicine and General Teaching HospitalPrague, Czech Republic; 2 Department of PediatricsHSK-Klinik, Wiesbaden, Germany; 3 Department of Pediatrics and Child Development, Universitäts-KinderklinikTübingen, Germany; 4 Formerly from Department of Genetic Medicine, Women's and Children's HospitalAdelaide, Australia

**Keywords:** sphingolipid activator proteins, prosaposin, urinary lipids, mass spectrometry, *PSAP* gene, saposin deficiency, metachromatic leukodystrophy

## Abstract

Prosaposin deficiency (pSap-d) and saposin B deficiency (SapB-d) are both lipid storage disorders caused by mutations in the *PSAP* gene that codes for the 65–70 kDa prosaposin protein, which is the precursor for four sphingolipid activator proteins, saposins A–D. We report on two new patients with *PSAP* gene defects; one, with pSap-d, who had a severe neurovisceral dystrophy and died as a neonate, and the other with SapB-d, who presented with a metachromatic leukodystrophy-like disorder but had normal arylsulfatase activity. Screening for urinary sphingolipids was crucial to the diagnosis of both patients, with electrospray ionization tandem mass spectrometry also providing quantification. The pSap-d patient is the first case with this condition where urinary sphingolipids have been investigated. Multiple sphingolipids were elevated, with globotriaosylceramide showing the greatest increase. Both patients had novel mutations in the *PSAP* gene. The pSap-d patient was homozygous for a splice-acceptor site mutation two bases upstream of exon 10. This mutation led to a premature stop codon and yielded low levels of transcript. The SapB-d patient was a compound heterozygote with a splice-acceptor site variant exclusively affecting the SapB domain on one allele, and a 2 bp deletion leading to a null, that is, pSap-d mutation, on the other allele. Phenotypically, pSap-d is a relatively uniform disease of the neonate, whereas SapB-d is heterogeneous with a spectrum similar to that in metachromatic leukodystrophy. The possible existence of genotypes and phenotypes intermediate between those of pSap-d and the single saposin deficiencies is speculated. © 2009 Wiley-Liss, Inc.

## INTRODUCTION

Prosaposin (pSap) is a non-enzymic 65–70 kDa glycoprotein encoded by the *PSAP* gene [Sandhoff et al., 2001]. Amongst its roles, pSap is the precursor for four saposins (Saps) A–D, which are formed by proteolysis. The Saps, also known as sphingolipid activator proteins, are indispensable cofactors for the intralysosomal degradation of a number of sphingolipids and seem to interact directly with the specific lipid hydrolases and/or facilitate presentation of the lipid substrates to these enzymes [Sandhoff et al., 2001; Spiegel et al., 2005]. Defects in the *PSAP* gene can cause a deficiency of either the entire pSap protein (prosaposin deficiency, pSap-d) or an individual Sap: SapA-d, SapB-d, SapC-d, or SapD-d, with, to date, SapD-d only being reported in an animal model [Matsuda et al., 2004]. In humans, pSap-d is a unique neonatal condition with an acute generalized neurovisceral dystrophy associated with the storage of multiple sphingolipids, whereas each isolated Sap deficiency is generally similar to a particular sphingolipid hydrolase-deficiency, namely, SapA-d to Krabbe leukodystrophy [Spiegel et al., 2005], SapB-d to metachromatic leukodystrophy (MLD), and SapC-d to Gaucher disease [Sandhoff et al., 2001]. The pathologies and biochemical phenotypes observed in pSap-d and the single Sap-deficient diseases have provided indirect insight into the specific roles and normal functions, including certain neurotrophic effects, of p-Sap and/or the individual Saps.

We report on two additional patients, one with pSap-d and the other with SapB-d. Both patients were detected by urinary glycosphingolipid analysis and they also have novel *PSAP* mutation(s). Tandem mass spectrometry (MS/MS) of the urinary lipids proved to be an efficient screening method. The distinctive pattern found in urine from the present pSap-d patient, with elevations in multiple sphingolipids, including ceramide, constitutes the first urine sphingolipid analysis for this condition.

## PATIENTS AND METHODS

### Patient 1

**FIG. 1:**
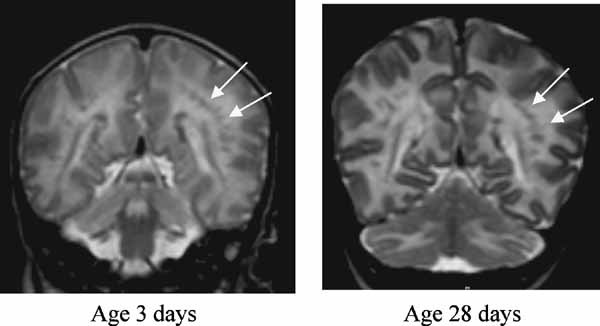
Cranial MRI scans (T2 weighted) for patient 1 (pSap-d). Note the absence of gyri cinguli and the corpus callosum, which is thinned. The arrows point to dark lesions arranged in a chain-like manner that are suggestive of gray matter heterotopias.

**FIG. 2:**
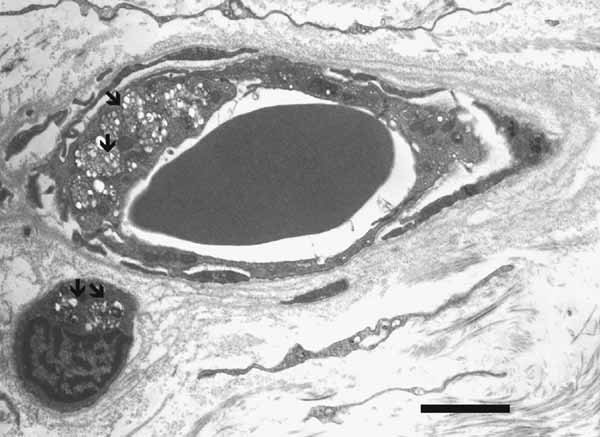
Skin biopsy from patient 1 (pSap-d). Arrows mark clusters of heterogeneous lysosomal storage material containing bright (electron-lucent) vesicles in an endothelial cell (capillary vessel, upper half) and a perivascular cell (bottom left hand corner). Bar at bottom, 3 µm.

**FIG. 3:**
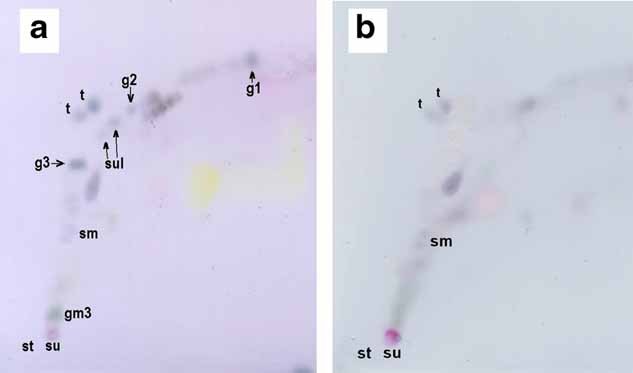
Urinary lipids in (**a)** patient 1 (pSap-d; 44-day-old), and (**b)** a normal control infant (11-month-old, with similarly low urinary creatinine as in patient 1) analysed by two-dimensional TLC (direction of first solvent [chloroform/methanol/water 14:6:1, by volume] was upward, and of the second solvent [chloroform/methanol/conc. acetic acid/water 40:8:1:1] from left to right). Lipids from each 3 ml urine sample were separated on silica-gel HPTLC plates and stained with anisaldehyde/sulfuric acid. Symbols (alphabetic): *g1*, glucosylceramide; *g2*, dihexosylceramides; *g3*, globotriaosylceramide; *gm3*, G_M3_ ganglioside (preliminary identification); *sm*, sphingomyelin; *st*, start of sulfatide test; *su*, start of urinary lipid extract; *sul*, patient sulfatide (double spot); *t*, sulfatide test (double spot, different fatty acid type as compared to *sul*) calibrated to an amount equivalent to 10-fold of the mean normal sulfatide amount in 3 ml urine from 1- to 10-year-old children (n = 5). Note the intense spots for *g1*, *g3*, and *gm3* in (a). [Color figure can be viewed in the online issue, which is available at http://www.interscience.wiley.com.]

Patient 1 was born at term (weight, 3.2 kg [P50]; length, 50 cm [P50]; occipital–frontal circumference [OFC], 32 cm [1.5 cm below P10]) after an uneventful pregnancy to parents who were first cousins. The mother had noticed frequent and rhythmic movements of the child in late pregnancy. Directly after birth he had precipitate movements and clonic fits that were resistant to anticonvulsive drugs. Sucking and swallowing were insufficient and tube feeding was started. After 3 weeks he had increased serum C-reactive protein and needed additional oxygen. A chest X-ray revealed pulmonary infiltrations. At the age of 4 weeks, he presented with muscle hypotonia, myoclonus and periods of twitching of the right arm and hand that were unresponsive to drugs. The liver and spleen were enlarged, which was confirmed by sonography, with the liver and spleen vertical diameters increased to 7 and 7.5 cm, respectively. Laboratory tests showed increased liver enzymes. Testing of white blood cell lysosomal enzymes revealed a very low galactosylceramide β-galactosidase (EC 3.2.1.46) activity. On ECG, there were signs of mitral insufficiency. In the eye fundi, the optic disks were atrophic, the right more so than the left, and the maculae were not demarcated. Sonography of the slightly microcephalic skull showed small ventricles and periventricular punctate echogenicities. On cerebral magnetic resonance imaging (MRI) a thin corpus callosum and bilateral absence of the gyrus cinguli were found; the periventricular white matter regions showed striking multiple symmetrical signal changes [similar to those reported in Elleder et al., 2005] suggestive of gray matter heterotopias (Fig. 1), although there was no complete iso-intensity with gray matter. An electroencephalogram (EEG) revealed general changes with invariant alpha-activity, multi-focal sharp waves, but no ictual patterns which were also absent when the child had clonic jerk sequences of the limbs and head. At the age of 5 weeks, cerebrospinal fluid analysis revealed an increased total protein (349 mg/dl; normal for age, 53 ± 22 [SD] mg/dl). In the smears of a bone marrow aspirate a few storage macrophage-like cells were seen. Electron microscopy of a skin biopsy revealed generalized lysosomal storage (Fig. 2). The child deteriorated and parenteral nourishment was necessary. Repeated pulmonary infections led to death at the age of 55 days (weight, 3.85 kg [0.35 kg below P10]; length, not recorded; OFC, 35.5 cm [2 cm below P10]). In a urinary sample collected at day 44 a number of glycosphingolipids were elevated on lipid thin layer chromatography (TLC) (Fig. 3). The complex sphingolipidosis suggested a diagnosis of **pSap-d**.

### Patient 2

Patient 2 was born abroad 2 weeks before term after an uneventful pregnancy. When he was 7 months old and had started crawling, the parents noticed that the movements of his right arm and leg were poor. When he was admitted at the age of 9 months, he had signs of a mild, right-sided, arm-accentuated spastic hemiparesis. Skull sonography revealed a left-temporal, large porencephalic cyst, and distension of the left-ventricle, suggesting a preceding infarction of the medial cerebral artery. An MRI scan confirmed the medial artery infarction, which probably occurred at or around the time of birth, and frontal sickle-shaped fluid pools were evident, suggesting retarded myelination. At the age of 12 months (weight, 10.5 kg [P50]; length, 80 cm [P75]; OFC, 47.5 cm [P50]), his spastic hemiparesis, with slight flexion of joints, did not prevent him from rolling over, although there was some hypotonia of the trunk muscles. At the age of 18 months his hemiparetic problems, with hand-fisting and accentuated patellar reflex on the right side, were moderate. He was able to reach a standing position. At the age of 23 months he had lost his previous ability to walk a few steps and was unable to stand freely or to crawl as actively as before. His limb muscles had distinctly reduced power. At 25 months of age he had muscle hypotonia, very weak peripheral reflexes and no Babinski sign. Laboratory values revealed an increased cerebrospinal fluid protein of 98 mg/dl (normal for age, 45 ± 15 mg/dl), but lactate was normal. At 28 months he was able to sit, stand, and play a little, but only with assistance. He often had periods of unmotivated crying. At 43 months he had his first generalized epileptic seizure, had lost his active speech and interest in toys, and had lower limb spasticity with a back-curved right knee. His hands were fisted and almost no longer used. His feet were held in the extensor position and he had a distinct spastic tetraparesis. A skull MRI scan revealed extensive white matter lesions with preserved U-fibers. Conduction velocity of the ulnar nerve was reduced to 12.7 m/sec (normal for age, >38 m/sec). Laboratory values showed normal activities of white blood cell lysosomal enzymes, including arylsulfatase A (ASA; EC 3.1.6.8) and galactosylceramide β-galactosidase (EC 3.2.1.46). Repeated infections, EBV positivity, and feeding problems were noted. At the age of 4 years, a preliminary diagnosis of MLD was made because of the finding of highly elevated urinary sulfatide. In view of the normal white blood cell ASA activity, **SapB-d** was suggested. Five months later the severely retarded, tetraspastic child had almost no eye contact with the examiner or fixation with the eyes. At the age of 5 years there was an additional tonic epileptic fit and the valproate dose was increased to 24 mg/kg body weight. At the age of 6 years (weight, 16.2 kg [P3]; length, 110 cm [P3]; OFC, 53 cm [P75]) generalized muscle hypertonicity, pes equinovarus, and eye pupils that were unreactive to light were noted.

### Urine Samples

Urine samples (24 hr collection) from patient 1 and 2 were kept frozen at −20°C. Frozen samples from Fabry patients, MLD patients, healthy children up to 12 years and adult controls were available.

### Enzyme Assays, Fibroblast Loading Tests, and Lipid Thin Layer Chromatography

The indicated methods were used for the determination of enzyme activities with radioactive substrates [Elleder et al., 2005], loading fibroblasts with radioactive glucosylceramide [Harzer et al., 1989], globotriaosylceramide [Schlote et al., 1991], sulfatide and sphingomyelin [Elleder et al., 2005], the TLC analysis of fibroblasts [Elleder et al., 2005], extraction of urine (please refer to supporting information S1 which may be found in the online version of this article), and TLC of urinary lipids [Schlote et al., 1991].

### Tandem Mass Spectrometry (MS/MS) Analysis of Urinary Sphingolipids

#### Preparation of lipid extracts for MS/MS analysis

Sonicated urine samples (150 µl) were extracted by mixing with 700 µl of a mixture of two volumes chloroform (no. 650471 Sigma–Aldrich Co., St. Louis, MO; grade CHROMASOLV® for HPLC 99.9%) to one volume methanol (no. 34966 Sigma–Aldrich; grade CHROMASOLV® for LC–MS Riedel-de Haën), containing a mixture of six internal standards (IST; for the sources, see later): 40 ng [C17:0]sulfatide, 35 ng [C17:0]GlcCer, 25 ng [C17:0]Gb3Cer, 50 ng [C17:0]ceramide, 50 ng [C16:0-D_3_]LacCer, and 50 ng [C17:0]sphingomyelin. The samples were vortexed for a few seconds at 5 min intervals three times. Then 150 µl Milli-Q® water was added and the mixing procedure repeated. After 30 min at room temperature the samples were centrifuged (14,500*g*, 5 min). Each separated lower phase was filtered (PTFE syringe pump filter) and re-washed by mixing with 500 µl Milli-Q® water [Han and Gross, 2005], the mixture centrifuged and separated as above. The final lower phases were evaporated under nitrogen. The dry residues were recovered with 500 µl chloroform–methanol 2:1 (v/v), and divided into 300 µl (positive ion mode) and 200 µl (negative ion mode) aliquots. The aliquots were evaporated under nitrogen. Before analysis the positive ion mode aliquot was dissolved in 300 µl 10 mM ammonium formate (no. 55674 Sigma–Aldrich; grade puriss. p.a. for LC–MS Fluka) in methanol [Liebisch et al., 1999; Fuller et al., 2005] and the negative mode aliquot in 200 µl methanol [Whitfield et al., 2001]. Lipids from cultured fibroblasts were extracted as described [Asfaw et al., 1998] with chloroform/methanol 2:1 (v/v) containing IST in the above-mentioned concentrations.

#### Electrospray ionization (ESI)-MS/MS analysis

The MS/MS equipment comprised an AB/MDS SCIEX API 3200 triple-quadrupole mass spectrometer (AB/MDS Sciex, Concord, Canada) with an ionspray source and an Agilent 1100 Autosampler (Agilent Technologies, Inc., Santa Clara, CA). Electrospray conditions and mass spectrometer ion optics were optimized for sphingolipid measurements using standard samples with lipid 10 µg/ml (for detailed conditions, see supporting information Table I which may be found in the online version of this article). Direct flow injection analysis, with methanol as the mobile phase, was done at a flow rate of 50 µl/min. Using the multiple reaction monitoring mode, the sphingolipids from a given sample were analyzed in series: For each sphingolipid, a 20 µl lipid extract aliquot corresponding to 6 µl urine, or to 0.2 µg fibroblast protein, was injected into the methanol mobile phase. Analysis was optimized for each sphingolipid, resulting in a sufficiently symmetrical peak shape with at least 12 measuring points per peak (e.g., see peaks in supporting information Fig. 1 which may be found in the online version of this article). This method allowed quantification of all major isoforms of each sphingolipid, distinguished by their fatty acid moiety (C16:0 to C26:0 fatty acids, non-substituted types and hydroxy-derivatives; details tabulated in supporting information Table II which may be found in the online version of this article). The negative ion mode was used for the analysis of sulfatide and the positive ion mode for neutral glycosphingolipids, ceramide, and sphingomyelin.

Quantification of sulfatide, Gb3Cer, dihexosylceramides (including LacCer and digalactosylceramide), monohexosylceramides (mainly GlcCer), ceramide, and sphingomyelin was done by single point calibration with a standard lipid concentration of 600 ng/ml (external calibration standard) corrected by the signal ratio toward IST. The concentrations of standard lipids and individual IST (see above) in the external calibration point were within the previously determined linear range of 50 ng–7 µg lipid per ml. The concentrations of IST in the external calibration point and in the patient samples were the same [De Hoffmann and Stroobant, 2002]. Reproducibility of all measurements was 93% or higher. The Student's *t*-test was used to determine statistical significance. The following porcine lipid standards were used: Sulfatide (no. 131305P) and sphingomyelin (no. 860062P) were from Avanti Polar Lipids, Inc., Alabaster, AL and Gb3Cer (no. 1067) and ceramide (no. 1056) from Matreya LLC, Pleasant Gap, PA. LacCer (bovine, no. G3166) and GlcCer (from human Gaucher spleen, no. G9884) were from Sigma–Aldrich. The MS/MS method cannot differentiate between the glucose (e.g., in GlcCer) and galactose (e.g., in digalactosylceramide) moieties because of the same mass. Therefore, GlcCer and galactosylceramide, as well as LacCer and digalactosylceramide, were quantified as monohexosylceramides and dihexosylceramides, respectively. Chemical identity and purity of sphingolipids used as calibration standards and as IST were proved by high performance TLC (HPTLC) and mass spectrometry (e.g., see supporting information Fig. 2 which may be found in the online version of this article). Purity of all sphingolipid standards was >97%.

#### Lipid internal standards

[C17:0]Gb3Cer (no. 2923.90), C16:0-D_3_-LacCer (no. 1534), and [C17:0]sphingomyelin (no. 1890), were from Matreya, and [C17:0]ceramide (no. 860517P) from Avanti. Internal standards which were not commercially available, [C17:0]sulfatide and [C17:0]GlcCer, were prepared from lyso-sulfatide (no. 573755, Calbiochem-Novabiochem GmbH, Schwalbach, Germany) and lyso-GlcCer (no. 1306, Matreya) by linking the lyso-compounds with C17:0 fatty acid (no. 10-1700-13, Larodan, Malmø, Sweden) using enzymatic semisynthesis with sphingolipid ceramide *N*-deacylase from *Pseudomonas* sp. TK4 (no. TAK 4462, Takara Shuzo, Shiga, Japan) as described [Mills et al., 2002]. The homogeneity of commercial and synthesized standards was confirmed by HPTLC and their identity by ESI-MS/MS.

### Molecular Analysis

Genomic DNA and total mRNA were isolated from the patients' cultured fibroblasts and from the parents' peripheral leukocytes by standard techniques. The *PSAP* gene was analyzed for mutations as described previously [Hůlková et al., 2001; Elleder et al., 2005]. Briefly, all coding exons and intron–exon boundaries were amplified from genomic DNA and sequenced directly from gel-purified PCR products using automated fluorescent sequencers. The mRNA was reverse-transcribed using Superscript II (Gibco®) and oligo-dT. RT-PCR products were sequenced as described above. Sequences were numbered sequentially from the A of the first ATG codon, which was designated 1 (reference genomic sequence: GenBank [http://www.ncbi.nlm.nih.gov/], **NC_000010.9**; reference mRNA sequence: GenBank, **NM_002778.1**; protein sequence: UniProtKB/Swiss-Prot [http://www.expasy.org/sprot/], **P07602** [SAP_HUMAN]).

## RESULTS

### Electron Microscopic Findings in Skin Biopsy

Confirming earlier results by A. Bornemann (Tübingen, personal communication), there was generalized dermal lysosomal storage expressed in the eccrine glands, capillary endothelium (Fig. 2), perivascular macrophages, Schwann cells, adipocytes, and some fibroblasts of patient 1. The storage lysosomes were around 1 µm in diameter and were filled with pleiomorphic, predominantly multivesicular structures; individual vesicles were around 170 nm in diameter.

### Biochemical Findings in Cultured Skin Fibroblasts

Fibroblast homogenates from patient 1 had a partially reduced activity of glucosylceramide β-glucosidase (EC 3.2.1.45), a more markedly reduced activity of galactosylceramide β-galactosidase, but a normal sphingomyelinase (EC 3.1.4.12) activity (for quantitative data, see supporting information S6 which may be found in the online version of this article). The activity of galactosylceramide β-galactosidase was normal in fibroblast homogenates from patient 2 (glucosylceramide β-glucosidase and sphingomyelinase not tested).

Thin layer chromatographic analysis of lipids extracted from fibroblasts (corresponding to about 0.5 mg protein) of patient 1 revealed intensely stained spots for ceramide, GlcCer, and LacCer similar to [Elleder et al., 2005]; other glycosphingolipids were not studied. The corresponding spots on chromatograms from control cells were about 3- to 10-fold less intense. Ceramide was additionally quantified by MS/MS and an amount of 16.2 µg/mg fibroblast protein found for patient 1 (normal range [n = 4], 3.6–6.2 µg/mg). A three to fourfold increase in dihexosylceramide and Gb3Cer concentrations in fibroblasts was also confirmed by MS/MS. Of these lipid elevations, only one was found to occur also in fibroblasts of patient 2: There was a fourfold increase in Gb3Cer.

Metabolic experiments with radioactive sphingolipid substrates (tritium-labeled on their ceramide moieties) loaded onto living fibroblast cultures from patient 1 and patient 2 gave similar results to those described for an earlier pSap-d [Elleder et al., 2005] and an earlier SapB-d [Schlote et al., 1991] patient, respectively. In fibroblasts from patient 1 there was an impaired turnover of [^3^H]ceramide (derived from loaded [^3^H]glucosylceramide or [^3^H]sphingomyelin). For cells from both patient 1 and patient 2, there was a reduced turnover of loaded [^3^H]globotriaosylceramide and [^3^H]sulfatide (supporting information S7 may be found in the online version of this article).

### Urinary Lipid Findings by Thin Layer Chromatography

Based on comparison with the staining intensity of lipids on the chromatogram from a control urine sample, the chromatogram for patient 1 (pSap-d) showed markedly increased levels of Gb3Cer, GlcCer, and G_M3_ ganglioside (preliminary identification), and sulfatides and dihexosylceramides were also elevated (Fig. 3). Sulfatides were also clearly elevated in the urine extract from patient 2 (SapB-d), and there was a slight increase in Gb3Cer.

### Urinary Lipid Findings by MS/MS

Table I summarizes the quantitative urinary sphingolipid findings in patients 1 (pSap-d) and 2 (SapB-d), as compared to findings in Fabry disease, MLD, and normal controls. The data were standardized relative to the concentration of sphingomyelin as a reference cellular sphingolipid. Lipid marked with footnotes c and e indicate that there was a statistically significant elevation when compared to the appropriate control group (infantile/late infantile controls for pSap-d, SapB-d, and MLD; adult controls for Fabry disease).

**TABLE I:** Urinary Sphingolipids in Patient 1 (pSap-d), Patient 2 (SapB-d), and in Pathologic and Normal Controls (ESI-MS/MS Determination)

	Lipid values^a^ expressed as µg/100 µg sphingomyelin				
	
	Sulfatide	Globotriaosylceramide	Lactosyl- and digalactosylceramide	Monohexosylceramide (mainly glucosylceramide)	Ceramide
Patient 1 (pSap-d) 44-day-old	67^c^	208^c^	45^c^	26^c^	17^c^
Patient 2 (SapB-d) 50-month-old	145^c^	51^c^	35^c^	14^c^	6.3
Metachromatic leukodystrophy 1- to 5-year-old (n = 6)	120^c^ ± 38^d^	8.8 ± 3.3	14 ± 5.3	6.2 ± 1.7	3.8 ± 1.1
Fabry disease males 24- to 54-year-old (n = 10)	6.8 ± 2.5^d^	201^c^ ± 102	35^e^ ± 19	3.5 ± 1.4	4.8 ± 1.7
Infantile/late-infantile controls 0.5- to 12-year-old (n = 16)	14 ± 5.2^d^	15 ± 8.2	10.2 ± 3.0	4.4 ± 1.0	4.3 ± 1.8
Adult controls males and females^b^ 17- to 60-year-old (n = 12)	9.7 ± 2.5^d^	21 ± 14	16 ± 6.1	4.4 ± 1.4	5.8 ± 2.8

a Mean of three determinations for patients 1 and 2. For the analytical reproducibility, see Patients and Methods Section.

b Fabry carrier status was excluded in control females molecularly.

c Statistical significance *P* < 0.001.

d Standard deviation.

e Statistical significance *P* < 0.01.

In patient 1, the abundance of Gb3Cer was elevated (*P* < 0.001) to a similar extent to that found in Fabry disease patients (Table I). However, patient 1 also showed additional elevations in sulfatide (*P* < 0.001) and the LacCer/digalactosylceramide (*P* < 0.001), monohexosylceramide (including GlcCer; *P* < 0.001), and ceramide (*P* < 0.001) fractions.

In patient 2, the elevated concentration of sulfatide (*P* < 0.001) was of a similar magnitude to that found in MLD (Table I). The distinct increase in the concentration of Gb3Cer (*P* < 0.001) was also remarkable.

The high elevations of the two urinary marker glycolipids, Gb3Cer, and sulfatide, had different proportions in patients 1 and 2: Gb3Cer and sulfatide concentrations accounted for 60% and 20% of excreted glycosphingolipids in patient 1, respectively, while they accounted for 21% and 59% in patient 2.

**FIG. 4:**
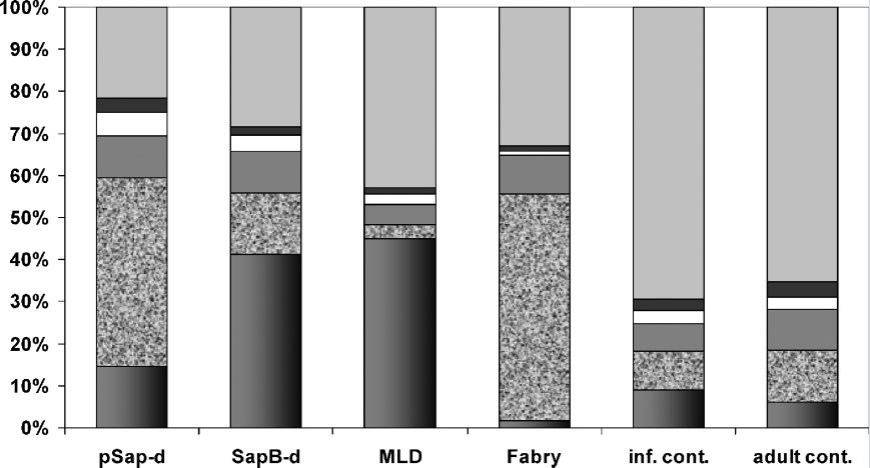
Percent distribution of main urinary sphingolipids. Order of column sections from bottom to top: sulfatide (right edge dark), globotriaosylceramide (sand-like), dihexosylceramides (gray), glucosylceramide (light), ceramide (black), sphingomyelin (light gray). Total analysed lipids were about 2–8 mg/L urine. Columns: *pSap-d*, patient 1; *SapB-d*, patient 2; *MLD*, metachromatic leukodystrophy group (mean, Table I); *Fabry*, Fabry disease group (mean, Table I); *inf. cont*., infantile/late-infantile controls (mean, Table I); *adult cont*., adult controls (mean, Table I).

In Figure 4, the percent distribution of concentrations of the main urinary sphingolipids is shown. This format, which allowed for a simple standardization of urinary lipid values in the absence of reference parameters, confirmed most of the findings summarized in Table I. The combined percentages for sulfatide, Gb3Cer, dihexosylceramides, glucosylceramide, and ceramide was higher in the diseases studied than for controls, with pSap-d having the highest percentage, consistent with the unique urinary multiple sphingolipid elevations in this condition.

### PSAP Gene Analysis

#### Patient 1 (pSap-d)

Patient 1 was found to be homozygous for a point mutation, c.1006-2A > G, in the *PSAP* gene. The mutation is located two bases upstream of the exon 10 acceptor splice site and alters the consensus splice site sequence. Analysis of the patient's cDNA sequence identified a splicing error with activation of a cryptic splice site in intron 9 leading to both an insertion of 70 bases from the intronic sequence into the mRNA (r.1006-70_1006-lins) and a premature stop codon. Each of the parents was found to be a carrier for the mutation. While it was possible to amplify the mutant mRNA by RT-PCR in the patient, the same analysis in the parents showed that, in comparison to the wild-type transcript, only trace amounts of the mutant transcript were present.

#### Patient 2 (SapB-d)

Patient 2 was found to be heterozygous for two mutations. The first mutation, c.577-2A > G, is a splicing mutation located in the acceptor splice site of intron 5. The second mutation is a 2bp deletion, c.828-829delGA, located in exon 8. The latter mutation leads to a frameshift and a premature stop codon. No transcript corresponding to the c.828-829delGA sequence was detected by RT-PCR in the patient. As this mutation leads to a premature stop codon, the most likely explanation for the absence of transcript is nonsense-mediated decay. Two transcripts from the other allele, which carries the c.577-2A > G, were found. Analysis of these indicated that the acceptor splice site in intron 5 was rendered non-functional by the mutation, with either of two different downstream acceptor sites being used instead. In the first transcript a cryptic splice site in exon 6 was used, since the mRNA contained a deletion of 21 bp from exon 6 (15 of them encoding SapB; r.577_597del). In the second transcript the whole of exon 6 was deleted (r.577_720del) and the exon 7 acceptor splice site was used. These in-frame deletions affected only the SapB domain, while the sequence encoding the remaining Saps was intact and in frame. Analysis of the parental genomic DNA showed that the mother was a carrier of c.577-2A > G sequence variant while the father was heterozygous for c.828-829delGA.

## DISCUSSION

Disorders caused by defects in the *PSAP* gene [Sandhoff et al., 2001] form a poorly known sub-group of lysosomal lipid storage diseases that are clinically and metabolically highly variable. Reports on the few known cases of pSap-d [Harzer et al., 1989; Bradová et al., 1993; Hůlková et al., 2001; Millat et al., 2003; Elleder et al., 2005] have indicated that this disorder should be considered in the differential diagnosis of neonates with unexplained neurologic signs, in particular, if these are combined with visceral involvement. The central nervous system changes in pSap-d may be caused not only by early lipid storage, but also by primary deficits in the organization of cerebral architecture, since pSap and/or some of its products are known to have essential neurotrophic functions. The present pSap-d patient was clinically and biochemically very similar to the earlier reported pSap-d patients [Harzer et al., 1989; Bradová et al., 1993; Hůlková et al., 2001; Millat et al., 2003; Elleder et al., 2005].

The diagnosis in the present SapB-d patient was complicated by the early finding of a massive infarction of the left arteria cerebri media, presumably coincidental to the leukodystrophy. However, the finding of high urinary sulfatide eventually led to a molecular diagnosis of SapB-d. This patient was similar to earlier reported patients [e.g., Schlote et al., 1991; Henseler et al., 1996]. Descriptions of other patients [Hahn et al., 1982; Rafi et al., 1992] suggest a highly variable clinical phenotype in SapB-d, similar to that in ASA–deficient MLD.

Urine is an accessible and non-invasive sample that can also be considered to be an “indirect kidney biopsy” for lipid and other analyses, due to the presence of portions of kidney cells [Desnick et al., 1970]. Solid materials from desquamated renal tubule epithelial and glomerular cells seem to provide the main source of urinary sphingolipids and other lipids, although some contribution from blood cells and plasma cannot be excluded, for example, in patients with kidney disease.

For patient and control urine samples, the proportion of each of the main sphingolipids relative to the total concentration of these sphingolipids can be calculated without standardization to a reference parameter (Fig. 4). In normal controls sphingomyelin accounted for more than 60% of these sphingolipids, but in the patients, the proportion of sphingomyelin was considerably less due to the preponderance of other sphingolipids. The relative proportions of individual urinary sphingolipids were, in general, diagnostically informative. However, the quantitative lipid signals from the mass spectrometric sphingolipid analysis of urine extracts should be standardized to a reference parameter (supporting information S8 may be found in the online version of this article). We used the ratio of signals for the different sphingolipids, including ceramide, to the urinary sphingomyelin concentration (Table I), in analogy to a described approach [Berná et al., 1999].

In this study we have demonstrated for the first time the use of urinary sphingolipid analysis when diagnosing the rare pSap-d condition. We have also confirmed the usefulness of this procedure when screening for SapB-d and other sphingolipidoses (Table I). In particular, urinary lipid analysis by ESI-MS/MS for the present pSap-d neonate verified the complex urinary lipid changes in this condition and allowed quantification of individual sphingolipid classes. There was a large increase in the concentration of Gb3Cer (within the range seen in adult Fabry disease) along with increases in sulfatide, dihexosylceramides (LacCer and digalactosylceramide), GlcCer, and ceramide. On the other hand, ESI-MS/MS analysis in the new patient with SapB-d showed a urinary concentration of sulfatide similar to that in MLD, and an elevated concentration of Gb3Cer, though lower than in adult Fabry disease males (see also supporting information S9 which may be found in the online version of this article). The urinary concentration of LacCer/digalactosylceramide was also increased in the SapB-d patient (Table I). Digalactosylceramide is a substrate that, like Gb3Cer, is thought to depend on intact SapB for its degradation by α-galactosidase A (EC 3.2.1.22) [Bradová et al., 1993] and, therefore, would be expected to be increased in SapB-d. Other authors [Li et al., 1985] have also reported elevated levels of this dihexosylceramide (in addition to LacCer, Gb3Cer, and sulfatide) in urine from SapB-d patients.

The c.1006-2A > G splice-site mutation in patient 1 (pSap-d) results in a premature stop codon with, when compared to the wild-type transcript, only traces of the mutant transcript detected by RT-PCR, indicating that it was probably largely removed through nonsense-mediated decay. Consistent with this view, another premature stop codon mutation located downstream of the SapB domain, as in the present patient, also results in an apparently mRNA-negative (pSap-d) allele [Diaz-Font et al., 2005]. Although we could not completely exclude the possibility that the residual mutant transcript gave rise to small amounts of functional SapA and SapB in our patient in vivo, the patient's clinical and biochemical phenotype was as severe as in previously described patients, supporting the view that functional SapA and SapB were absent.

The two mutations carried by patient 2 (SapB-d) affect the fate of the prosaposin transcript in very different ways. A 2 bp deletion (c.828-829delGA) in exon 8 on one allele results in complete absence of the transcript, most likely due to nonsense-mediated decay, so no pSap or Saps would be generated from this allele. On the other allele, the c.577-2A > G splicing mutation leads to the formation of two alternative transcripts, both of which carry an in-frame deletion of a portion of the SapB domain. This mutation exclusively affects the SapB domain and should not result in the deficiency of other Saps. In keeping with this finding, the biochemical and also clinical phenotype in this patient were consistent with an isolated absence of SapB.

To date, four different genotypes (all homozygous), including the one for the present patient, have been reported for pSap-d [Schnabel et al., 1992; Hůlková et al., 2001; Millat et al., 2003]. All have led to a complete loss of functional pSap and Saps and a rather uniform phenotype. In contrast, for the SapB-d condition not only late infantile but also later manifesting, even adult, phenotypes [Hahn et al., 1982; Rafi et al., 1992] have been described. SapC-d is similar to SapB-d in this respect, with patients presenting with a range of phenotypes including neuronopathic and non-neuronopathic forms [Tylki-Szymańska et al., 2007]. In deficiencies of single Saps the second allele can be a null (pSap-d) allele, with the three active Saps derived from only one allele (refer to [Diaz-Font et al., 2005; Tylki-Szymańska et al., 2007] for examples of SapC-d and the patients described in [Holtschmidt et al., 1991; Deconinck et al., 2008] and the present patient for examples of SapB-d).

However, other mutant *PSAP* genotypes may occur which affect the function of more than one Sap domain but less than the whole pSap. In a two-saposin-deficient (Saps C and D inactive) mouse model, the resulting phenotype [Sun et al., 2007] included some but not all features of pSap-d mice [Fujita et al., 1996]. One 28-month-old patient [Wenger et al., 1989] was described to have SapB-d, but was not studied molecularly. Of note, his clinical and pathologic findings indicated a generalized neurovisceral dystrophy comparable to that in pSap-d, in addition to symptoms suggestive of MLD. The study of additional mutant *PSAP* genotypes and phenotypes should contribute to the better understanding of the functions of the pSap/Saps system.

## Abbreviations used

ASA: arylsulfatase A (EC 3.1.6.8)
ESI-MS/MS: electrospray ionization tandem mass spectrometry
Gb3Cer: globotriaosylceramide
GlcCer: glucosylceramide
HPTLC: high performance thin layer chromatography
IST: internal standards
LacCer: lactosylceramide
MLD: metachromatic leukodystrophy
MS/MS: tandem mass spectrometry
OFC: occipital–frontal circumference
pSap: prosaposin
pSap-d: prosaposin deficiency
Sap(s), saposin(s) including SapA, SapB, SapC: SapD
SapA-d, SapB-d, SapC-d, SapD-d: deficiency of the respective Sap
TLC: thin layer chromatography
