# Supplementary material for: Prosaposin Deficiency and Saposin B Deficiency (Activator-Deficient Metachromatic Leukodystrophy): Report on Two Patients Detected by Analysis of Urinary Sphingolipids and Carrying Novel PSAP Gene Mutations
Source: Am J Med Genet A. 2009 Mar 6;149A(4):613–21. doi: 10.1002/ajmg.a.32712 (PMC3437469; doi:10.1002/ajmg.a.32712)
Supplement: Supplementary file 1 [file ajmg149A-0613-SD1.pdf]

Supplementary Materials to the Paper entitled

Prosaposin Deficiency and Saposin B Deficiency (Activator-Deficient Metachromatic Leukodystrophy); Report on Two Patients Detected by Analysis of Urinary Sphingolipids and Carrying Novel PSAP Gene Mutations.

(American Journal of Medical Genetics, vol. ….)

Supplementary material S1:

For thin layer chromatography, urinary lipids were extracted as follows: First, 3 ml urine was acidified with 10 µl concentrated hydrochloric acid and left at 6° C for 10 h. The sample was then mixed with 9 ml methanol and 8 ml chloroform and phase-partitioned with 5 ml water. The clear portion of the upper phase was removed and the lower phase partitioned twice more in the same way. The final lower phase was dried and the residue re-extracted with 1 ml chloroform/methanol 2:1 (v/v). The extract was concentrated 20-fold and then applied as a spot to the lower left corner of a silica gel HPTLC plate (no. 1.05631, Merck, Darmstadt, Germany) for two-dimensional separation.

Supplementary material S2 (= supplementary Table 1):

Supplementary Table 1 - Conditions of ion source and quadrupole section of mass analyzer

	**Ceramide**	**Monohexosyl-ceramides**	**Lactosyl- & digalactosyl-ceramide**	**Globo-triaosyl-ceramide**	**Sphingo-myelin**	**Sulfatide**
**Courtain gas (psi)**	**10**	**10**	**10**	**10**	**10**	**10**
**Voltage (kV)**	**4.5**	**4.5**	**4.5**	**4.5**	**4.5**	**-4.5**
**Temperature (^{o}C)**	**200**	**200**	**200**	**200**	**200**	**200**
**Source gas 1 (psi)**	**20**	**20**	**20**	**20**	**20**	**20**
**Source gas 2 (psi)**	**55**	**55**	**55**	**45**	**55**	**45**
**Declustering potential (V)**	**60**	**47**	**65**	**78**	**76**	**-150**
**Entrance potential (V)**	**5**	**4.9**	**6**	**7.9**	**5.6**	**-9**
**Collision energy (V)**	**42**	**48**	**64**	**74**	**37**	**-130**
**Collision gas (psi)**	**5**	**5**	**5**	**3**	**5**	**3**
**Collision cell exit potential (V)**	**5.7**	**5.6**	**5.6**	**5.4**	**4.1**	**-1**

Nitrogen was used as the collision gas

Supplementary material S3 (= supplementary Fig. 1):

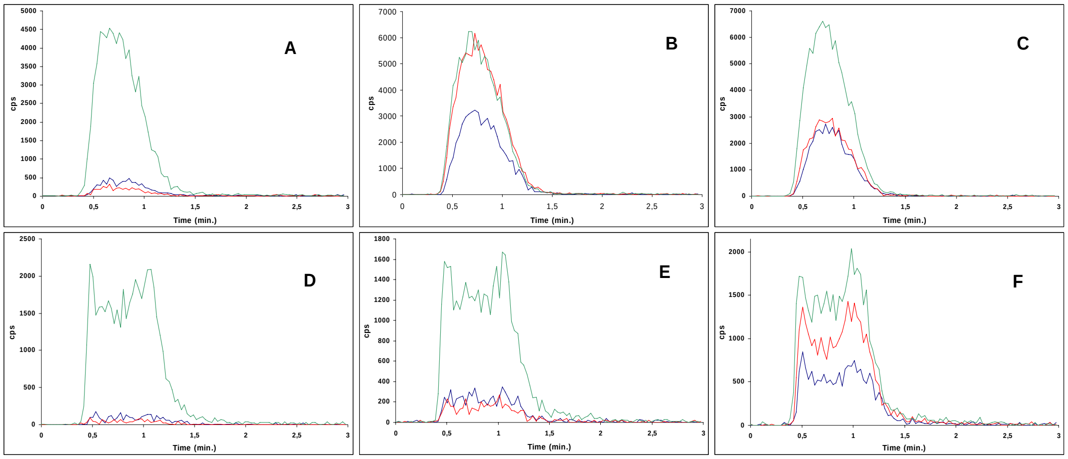

Supplementary Fig. 1: Examples of extracted ion current of MS/MS measurements of selected isoforms of sphingolipids (sulfatides and monohexosylceramides [MHC]) in urine. Abscissa = time of measurement in minutes, ordinate = signal intensity in counts per second (cps). A – sulfatide, infantile control; B – sulfatide, patient 2 (SapB-d); C – sulfatide, patient 1 (pSap-d); D – MHC, infantile control; E – MHC, patient 2 (SapB-d); F – MHC, patient 1 (pSap-d). Different colors represent specific lipid isoform as follows: blue for C16:0 isoforms (sufatide and MHC), red for C24:0 (sulfatide) or C24:1 (MHC) isoforms. Green is for ISTs ([C17:0]sulfatide or [C17:0]MHC). Differences in concentration of lipids are evident when comparing signals of urinary isoforms to signals of internal standards in controls and in patients. Please note that in the infantile control samples (A and D) the signals were also sufficiently high to be measured.

Supplementary material S4 (=supplementary Table 2):

Supplementary Table 2 – Transition ion pairs for MRM measurement and quantitation of sphingolipids

Ceramide			
**Isoform by fatty acid**	**Parent ion**	**Daughter ion**	**Scan time**
**C14:0**	**510.7**	**264.4**	**100 ms**
**C16.0**	**538.8**	**264.4**	**100 ms**
**C18:0**	**566.8**	**264.4**	**100 ms**
**C18:0-OH**	**582.8**	**264.4**	**100 ms**
**C20:0**	**594.9**	**264.4**	**100 ms**
**C22:0**	**622.8**	**264.4**	**100 ms**
**C22:1-OH**	**636.9**	**264.4**	**100 ms**
**C24:0**	**650.8**	**264.4**	**100 ms**
**C24:1**	**648.8**	**264.4**	**100 ms**
**C24:2**	**646.8**	**264.4**	**100 ms**
**C26:0**	**678.8**	**264.4**	**100 ms**
**C26:1**	**676.8**	**264.4**	**100 ms**
**C26:2**	**674.8**	**264.4**	**100 ms**
**C24:0-OH**	**666.7**	**264.4**	**100 ms**
**C24:1-OH**	**664.7**	**264.4**	**100 ms**
**C24:2-OH**	**662.7**	**264.4**	**100 ms**
**C26:0-OH**	**694.7**	**264.4**	**100 ms**
**C26:1-OH**	**692.7**	**264.4**	**100 ms**
**C22:0-OH**	**638.8**	**264.4**	**100 ms**
**C17:0 (internal standard)**	**552.6**	**264.4**	**100 ms**
Monohexosylceramides			

**Isoform by fatty acid**	**Parent ion**	**Daughter ion**	**Scan time**
**C16:0**	**700.8**	**264.4**	**100 ms**
**C18:0**	**728.8**	**264.4**	**100 ms**
**C20:0**	**756.9**	**264.4**	**100 ms**
**C22:0**	**784.9**	**264.4**	**100 ms**
**C22:1**	**782.9**	**264.4**	**100 ms**
**C22:1-OH**	**798.9**	**264.4**	**100 ms**
**C24:0**	**812.9**	**264.4**	**100 ms**
**C24:1**	**810.9**	**264.4**	**100 ms**
**C24:2**	**808.9**	**264.4**	**100 ms**
**C24:0-OH**	**828.9**	**264.4**	**100 ms**
**C24:1-OH**	**826.9**	**264.4**	**100 ms**
**C24:2-OH**	**824.8**	**264.4**	**100 ms**
**C26:0**	**840.9**	**264.4**	**100 ms**
**C26:1**	**838.9**	**264.4**	**100 ms**
**C26:2**	**836.7**	**264.4**	**100 ms**
**C26:0-OH**	**856.9**	**264.4**	**100 ms**
**C17:0 (internal standard)**	**714.5**	**264.4**	**100 ms**
Lactosyl- and digalactosylceramide			
**Isoform by fatty acid**	**Parent ion**	**Daughter ion**	**Scan time**
**C16:0**	**862.7**	**264.4**	**100 ms**
**C18:0**	**890.7**	**264.4**	**100 ms**
**C20:0**	**918.7**	**264.4**	**100 ms**
**C22:0**	**946.8**	**264.4**	**100 ms**
**C22:1**	**944.8**	**264.4**	**100 ms**
**C22:1-OH**	**960.8**	**264.4**	**100 ms**
**C24:0**	**974.9**	**264.4**	**100 ms**

**C24:1**	**972.9**	**264.4**	**100 ms**
**C24:2**	**970.9**	**264.4**	**100 ms**
**C24:0-OH**	**990.9**	**264.4**	**100 ms**
**C24:1-OH**	**988.9**	**264.4**	**100 ms**
**C22:0-OH**	**962.6**	**264.4**	**100 ms**
**C16:0-D_3 (internal standard)**	**865.7**	**264.4**	**100 ms**
Globotriaosylceramide			
**Isoform by fatty acid**	**Parent ion**	**Daughter ion**	**Scan time**
**C16:0**	**1024.6**	**264.4**	**100 ms**
**C18:0**	**1052.7**	**264.4**	**100 ms**
**C18:1**	**1068.7**	**264.4**	**100 ms**
**C20:0**	**1080.8**	**264.4**	**100 ms**
**C22:0**	**1108.7**	**264.4**	**100 ms**
**C22:1**	**1106.8**	**264.4**	**100 ms**
**C22:0-OH**	**1124.8**	**264.4**	**100 ms**
**C22:1-OH**	**1122.7**	**264.4**	**100 ms**
**C24:0**	**1136.8**	**264.4**	**100 ms**
**C24:1**	**1134.8**	**264.4**	**100 ms**
**C24:2**	**1132.6**	**264.4**	**100 ms**
**C24:0-OH**	**1152.8**	**264.4**	**100 ms**
**C24:1-OH**	**1150.8**	**264.4**	**100 ms**
**C24:2-OH**	**1148.8**	**264.4**	**100 ms**
**C26:0**	**1164.8**	**264.4**	**100 ms**
**C26:1**	**1162.9**	**264.4**	**100 ms**
**C26:2**	**1160.8**	**264.4**	**100 ms**
**C26:0-OH**	**1180.9**	**264.4**	**100 ms**
**C26:1-OH**	**1178.6**	**264.4**	**100 ms**
**C26:2-OH**	**1176.7**	**264.4**	**100 ms**

**C17:0 (internal standard)**	**1038.7**	**264.4**	**100 ms**
Sphingomyelin			
**Isoform by fatty acid**	**Parent ion**	**Daughter ion**	**Scan time**
**C16:0**	**703.7**	**184.2**	**100 ms**
**C16:1**	**701.7**	**184.2**	**100 ms**
**C18:0**	**731.7**	**184.2**	**100 ms**
**C18:1**	**729.7**	**184.2**	**100 ms**
**C20:0**	**759.8**	**184.2**	**100 ms**
**C20:1-OH**	**773.8**	**184.2**	**100 ms**
**C22:0**	**787.8**	**184.2**	**100 ms**
**C22:1**	**785.8**	**184.2**	**100 ms**
**C24:0**	**815.8**	**184.2**	**100 ms**
**C24:1**	**813.8**	**184.2**	**100 ms**
**C24:2**	**811.8**	**184.2**	**100 ms**
**C22:0-OH**	**803.8**	**184.2**	**100 ms**
**C22:1-OH**	**801.8**	**184.2**	**100 ms**
**C24:0-OH**	**831.8**	**184.2**	**100 ms**
**C24:1-OH**	**829.8**	**184.2**	**100 ms**
**C24:2-OH**	**827.8**	**184.2**	**100 ms**
**C22:2-OH**	**799.9**	**184.2**	**100 ms**
**C17:0 (internal standard)**	**717.7**	**184.2**	**100 ms**
Sulfatide			
**Isoform by fatty acid**	**Parent ion**	**Daughter ion**	**Scan time**
**C16:0**	**778.6**	**97.0**	**100 ms**
**C18:0**	**806.7**	**97.0**	**100 ms**
**C18:0-OH**	**822.7**	**97.0**	**100 ms**
**C20:0**	**834.7**	**97.0**	**100 ms**

**C20:0-OH**	**850.7**	**97.0**	**100 ms**
**C22:0**	**862.8**	**97.0**	**100 ms**
**C22:1-OH**	**876.7**	**97.0**	**100 ms**
**C22:0-OH**	**878.7**	**97.0**	**100 ms**
**C24:1**	**888.8**	**97.0**	**100 ms**
**C24:0**	**890.7**	**97.0**	**100 ms**
**C23:0-OH**	**892.8**	**97.0**	**100 ms**
**C24:1-OH**	**904.8**	**97.0**	**100 ms**
**C24:0-OH**	**906.8**	**97.0**	**100 ms**
**C26:1**	**916.8**	**97.0**	**100 ms**
**C26:0**	**918.9**	**97.0**	**100 ms**
**C26:1-OH**	**932.8**	**97.0**	**100 ms**
**C26:0-OH**	**934.9**	**97.0**	**100 ms**
**C17:0 (internal standard)**	**792.7**	**97.0**	**100 ms**

Supplementary material S5 (= supplementary Fig. 2):

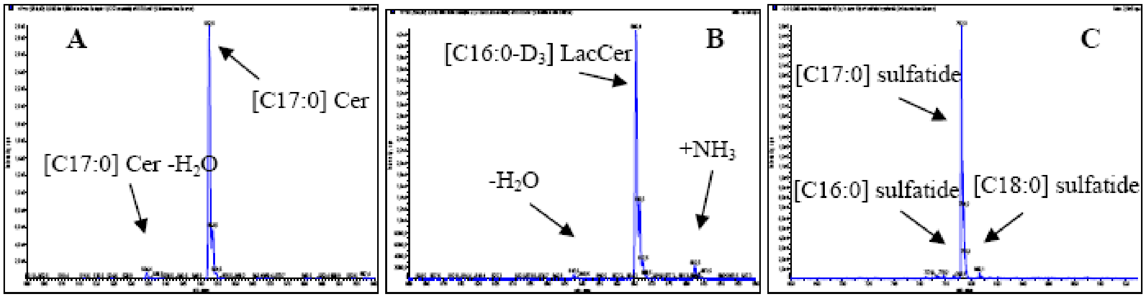

Supplementary Fig. 2: Chemical identity and isoform purity of selected sphingolipid standards: [C17:0]ceramide (A), [C16:0-D_3]lactosylceramide (B) and [C17:0]sulfatide (C). Abscissa = m/z (mass to charge ratio), Ordinate = signal intensity in cps. Precursor ion scans of the lipids used as internal standards were undertaken to prove their chemical identity. Precursor scan for ion with m/z 264.4 in positive ion mode was performed for ceramide and lactosylceramide (LacCer) and for ion with m/z 97 in negative ion mode for sulfatide. The ceramide and LacCer standard each revealed only a single major peak corresponding to the lipid isoform, with some minor peaks resulting from loss of water and, in the case of LacCer, formation of ammonia adducts, which can be explained as an effect of ammonium formate in methanol, also evident. Only traces of byproducts (up to 3%) were detected in [C17:0]sulfatide and consisted of [C16:0] and [C18:0] sulfatide isoforms probably generated during the enzymatic semisynthesis with fatty acids bound to the commercial enzyme preparation.

Supplementary material S6:

The following enzyme activities were found in fibroblast homogenates from patient 1: Glucosylceramide β–glucosidase (EC 3.2.1.45; ’low substrate assay’ [Elleder et al., 2005; for reference, see main paper]); mean of duplicates: 1.65 units (where 1 unit = 1 nmol radioactive substrate cleaved per h per 10^6 cells; normal range [n=5], 2.2 – 4.9 units) and galactosylceramide β–galactosidase (EC 3.2.1.46; mean of duplicates: 0.41; normal range, 1.3

– 3.8 units), but a normal sphingomyelinase (EC 3.1.4.12; mean of duplicates: 22.0; normal range, 7.7 – 27.8 units) activity.

Supplementary material S7:

Metabolic experiments with radioactive sphingolipid substrates (tritium-labelled on their ceramide moieties) loaded onto living fibroblast cultures from patient 1 and patient 2 gave similar results to those described for an earlier pSap-d [Elleder et al., 2005; for references, see main paper] and an earlier SapB-d [Schlote et al., 1991] patient, respectively. Loading fibroblasts with [^{3}H]glucosylceramide resulted in a higher retention of radioactivity within ceramide in cells from patient 1 (31 % of incorporated radioactivity) than in control cells (4 to 8 %), demonstrating a block at ceramide degradation. This block was also evident when cells from patient 1 were loaded with [^{3}H]sphingomyelin (70 % of radioactivity recovered in ceramide compared to 10 to 14 % in controls). For cells from both patient 1 and patient 2, there was a reduced turnover of loaded [^{3}H]globotriaosylceramide (3- to 5-fold lower radioactivity in LacCer and other metabolites) and of independently loaded [^{3}H]sulfatide (about 3-fold lower radioactivity in galactosylceramide and other metabolites) in comparison to controls, a finding similar to that seen with metachromatic leukodystrophy cells.

Supplementary material S8:

The widely used urinary creatinine concentration proved unsuitable as a reference parameter in our experience, possibly due to the physiological considerations mentioned above. In particular, when the urinary creatinine concentration was lower than 1 mmol per litre, the ratios of quantitative MS/MS lipid signals to creatinine levels often appeared to be falsely high. Others have used the ratio of signal to urinary phospholipids, e.g., urinary phosphatidyl choline concentration [Whitfield et al., 2001; for reference, see main paper], whereas we used a ratio of specific lipid signal/urinary sphingomyelin concentration.

Supplementary material S9:

The lipid abnormalities in urine could be qualitatively explained by the specific absences of Saps [Sandhoff et al., 2001; for references, see main paper] in pSap-d (patient 1) and SapB-d (patient 2). But it was unclear, why the ratio of Gb3Cer to sulfatide concentration was about 3 in pSap-d versus about 0.3 in SapB-d (for individual Gb3Cer and sulfatide values, see Table I in main paper). Given that SapB is required for the breakdown of both of these glycolipids [Sandhoff et al., 2001] and it is absent in pSap-d as well as SapB-d, additional factors seem to have contributed to this difference. One factor might have been the discordant ages at the time of study (pSap-d, 44-day-old; SapB-d, 50-month-old). However, in pSap-d the absence of SapA, C and D, in addition to that of SapB, may also have indirectly contributed to the high elevation of urinary Gb3Cer. Moreover, a role for the absence of SapD in pSap-d might be deduced from the findings in a SapD-d mouse model [Matsuda et al., 2004], where the striking degeneration of renal tubule cells might be viewed as a factor relevant to sphingolipid levels.

In addition, there were increased urinary concentrations of LacCer/digalactosalceramide, monohexosylceramide and ceramide in the SapB-d patient (Table I of main paper). These non-sulfatide lipid elevations might be explained as secondary effects of the high sulfatide abundance in renal cells. Moreover, LacCer sulfate (a minor component of sulphated glycolipids compared to the predominating galactosylceramide sulfate [= sulfatide, as discussed above]) was also found to be elevated in patient 1 and patient 2 (results not shown).
